# Conductive Polymer Composites for Hydrogen Sulphide Sensors Working at Sub-PPM Level and Room Temperature

**DOI:** 10.3390/s21196529

**Published:** 2021-09-30

**Authors:** Caroline Duc, Mohamed Lamine Boukhenane, Thomas Fagniez, Lahcen Khouchaf, Nathalie Redon, Jean-Luc Wojkiewicz

**Affiliations:** 1 IMT Nord Europe, Institut Mines-Télécom, University of Lille, Centre for Energy and Environment, F-59000 Lille, France; mohamed-lamine.boukhenane@imt-lille-douai.fr (M.L.B.); Thomas.fagniez@imt-nord-europe.fr (T.F.); nathalie.redon@imt-nord-europe.fr (N.R.); jean-luc.wojkiewicz@imt-nord-europe.fr (J.-L.W.); 2 IMT Lille Douai, Institut Mines-Télécom, University of Lille, Centre for Materials and Processes, F-59000 Lille, France; lahcen.khouchaf@imt-nord-europe.fr

**Keywords:** gas sensing, hydrogen sulphide, conductive polymer, resistive sensor, hybrid sensors

## Abstract

Hybrid composites based on tin chloride and the conductive polymers, polyaniline (PAni) and poly(3,4-ethylenedioxythiophene) polystyrene sulfonate (PEDOT:PSS), were integrated into high-performance hydrogen sulphide (H2S) gas sensors working at room temperature. The morphology and chemical properties were studied by scanning and transmission electron microscopy (SEM, TEM), energy dispersive spectroscopy (EDS) and Fourier-transform infrared (FTIR). The composites demonstrated a slightly porous nanostructure and strong interactions between the polymers and the metal salt, which slightly dopes PAni. The hybrid sensors exhibited a very low detection limit (<85 ppb), fast response, repeatability, reproducibility and stability over one month. Moreover, this work presents how calibration based on the derivative of the signal can give hybrid sensors the ability to quantify the concentration of targeted gas, even during continuous variation of the analyte concentration. Finally, the effect of interfering species, such as water and ammonia, is discussed.

## 1. Introduction

Malodorous and hazardous at a sub-ppm level, hydrogen sulphide (H_2_S) is a water-soluble gas that can lead to death by poisoning at higher concentrations [1]. Occupational Safety and Health Administration describes H_2_S as one of the most dangerous gases to workers, behind carbon monoxide [2]. Originating from natural and human sources, H_2_S is produced by geothermal activity (e.g., hot spring, natural gas, crude petroleum), organic decomposition (e.g., in tanneries, sewers, Sargassum algae, etc.) or synthetized intentionally for agricultural applications and sulphuric acid production [3]. Several exposure limits, from 1 ppb to 100 ppm, have been established by international public safety organizations in order to (i) limit olfactory disturbance and (ii) protect workers [1]. For these purposes, devices functioning in various concentration range and environmental conditions are needed.

Ordinarily, the quantification of H_2_S in air is ensured by highly sensitive analytical methods, such as spectrometry. Despite their high sensitivity at low gas levels, these methods present the disadvantages of high costs, low temporal resolution display and complex sampling and analytical processes [4,5]; cost-effective and easy-to-use apparatuses resulting in continuous real-time measurements are required for odor monitoring and safety control. Therefore, many studies have been performed to develop fast, user-friendly, portable and low-cost devices [6] in order to fulfil these requirements.

Among the most common H_2_S sensors (chemiresistive, electrochemical or optical) [7,8], the chemiresistive sensor is the simplest transducer. Based on the resistance measurement of a sensitive material in contact with two electrodes, it turns a chemical fluctuation into an electrical signal [9]. Given their high sensitivity, quick response and ease of integration in miniaturized electronic devices, metal oxides (MOx) are the predominant sensitive material in gas detection [10,11] and, more specifically, in H_2_S detection [12,13]. Nevertheless, MOx-based sensors offer a low selectivity, as well as a high sensitivity to relative humidity variation; and they generally operate at high temperatures (>100 °C).

Conductive polymers (CPs), such as polyaniline (PAni), represent a promising substitute for MOx [9]. Known to have a controllable conductivity by redox and acid/base reactions, PAni has been used in several gas sensing applications, of which the most common is related to the detection of ammonia [14].

Among the approaches used to make PAni selective towards H_2_S (e.g., the inclusion of metal nanoparticles, metal oxide or carbon-based nanostructures) [15], the addition of metal chloride has shown the highest response in the range 2.5 to 15 ppm, with changes in resistance about several orders of magnitude [16,17,18,19]. Indeed, in the presence of PAni, the metal chloride reacts with H_2_S gas to release a strong acid (HCl), which dopes the polymer and increases its conductivity [16]. Mainly based on the use of CuCl_2_, these devices show irreversibility and low repeatability given the fact that the reaction between the gas and the metal chloride leads to the formation of metal sulphide, which modifies the conductivity of the composite irreversibly [16,17,18,19].

This work studies the behavior of hybrid materials based on PAni, SnCl_2_ and poly (3,4-ethylenedioxythiophene) polystyrene sulfonate (PEDOT:PSS) toward H_2_S exposure at sub-ppm level. PAni and SnCl_2_ were used as a sensitizing agent and as an intermediate, respectively, to make PAni selective to H_2_S. Known for its high conductivity up to 500 S/cm [20], PEDOT:PSS was used to facilitate the charge transport in the material and to match the electronic properties of the hybrid with the resistance range required by the measurement set-up. Inspired by studies where the first-derivative of the response was used to facilitate the analysis of sensors [21,22], we show how the analysis of the derivative of the signal can overcome the irreversibility of the response. Beyond the high sensitivity, repeatability, reproducibility and the stability of the sensor over one month, the study highlights its ability to work under continuous variations of H_2_S. Finally, the effect of interfering species, such as water and ammonia, paves the path towards the optimization of these devices.

## 2. Materials and Methods

### 2.1. Materials

The tin (II) chloride (SnCl_2_, anhydrous 98%) was procured from Alfa Aesar (US). Sigma Aldrich (US) supplied the polyaniline emeraldine-base (PAni, average Mw~65,000), the dimethylformamide (DMF) and the ethylene glycol (EG). The aqueous solution of poly (3,4-ethylene dioxythiophene):polystyrene sulfonate (PEDOT:PSS) was procured from Heraeus (Germany, PH1000, PEDOT:PSS concentration 1–1.3% in water according to the manufacturer’s instructions). The calibrated cylinders of the H_2_S (10 ppm) and NH_3_ (10 ppm) gases (diluted in N_2_) were provided by Praxair (US) and by Messer (Germany), respectively.

### 2.2. Preparation of Samples

The first step consisted of the dispersal of the PAni in an organic solvent. Next, 40 mg of PAni-emeraldine base was added grain-by-grain to 5 mL of DMF, while the solvent was stored on a hot plate under magnetic stirring (60 °C, 400 rotation/minute). After 6 days of stirring at 60 °C, 62 mg of SnCl_2_ was added to the solution, leading to a slight change of color in the solution, from dark blue to greenish, indicating that the metal salt slightly dopes PAni [23,24].

Subsequently, in order to adjust the conductivity of the final samples, a solution of 20 mL of PEDOT:PSS with 1.23 g of EG was added in different proportions to the Pani-SnCl_2_ solutions in order to correspond to 1, 2, 3 and 4 wt% of the dry PEDOT:PSS in the solid material (Table S1 and Figure S1 in the Supplementary Information). After complete homogenization under magnetic stirring (30 min), the solutions were sonicated for 45 min, using an intermittent cycle (0.2/0.8 s, active/passive intervals,) at 80% of the maximal power of a Sonopuls HD 2070 sonicator (Bandelin).

Finally, the solutions were deposited on interdigitated electrodes and dried in an oven at 90 °C under vacuum (−0.9 bar) for 5 days. The interdigitated electrodes were provided by Synkera (US) and composed of golden lines deposited on a ceramic substrate. The conductive pad was 3 mm, with a gap and fingers of 140 µm (5 fingers per electrode).

The concentration of PEDOT:PSS (wt%) discussed below corresponds to the weight content of solid PEDOT:PSS in the dry composite.

### 2.3. Characterization of Material Properties

The electrical properties of the films were accessed using the Van Der Pauw method [25] on the Hall effect measurement system HMS-5300 from Ecopia. The samples came from solutions of polymer composites deposited on a polyimide foil (thickness 110 µm) and dried according to the same process as the sensors. Next, 5 × 5 mm^2^ squares were cut and microdots of silver ink were deposited to ensure ohmic contact with the probes. Three samples per formulation were considered. For each sample, the conductivity was calculated for three different nominal values of thickness (minimum, mean, maximum). The conductivity discussed in the following sections corresponds to the average value of the all measurements associated with each sample. Thus, all error types are included.

An optical profilometer STIL Micromesure 2 working with SPIPTM software (Scanning Probe Image Processor) was used to measure the thickness of the sensitive films. The thickness value used in the conductivity calculation (5–12 µm) corresponded to the average value of 10 measurements from different parts of the film.

For the Fourier-transform infrared (FTIR) analysis, the composites were deposited on silicon substrate and their spectra were recorded 7 days after their drying, in the range of 400–4000 cm^−1^ at 64 scans per spectrum, using a FTIR Spectrometer Nicolet iS5 equipped with iD5 ATR from Thermo Scientific. The spectral features of the silicon substrate were removed by using the absorption subtraction technique. The position of each of the bands was calculated from the mean of more than three spectra per sample (Table 1).

The morphology of the composites was characterized using Scanning Electron Microscopy on a Zeiss Ultra 55 (1 kV, working distance 5.1 mm) and Transmission Electron Microscopy (TEM) on a FEI Tecnai G2-20 instrument (200 kV) equipped with a filament of lanthanum hexaboride LaB6 and a Gatan digital camera CCD ORIUS. The elemental analysis was carried out using Energy-dispersive X-ray spectroscopy (EDS) from Bruker.

### 2.4. Characterization of Sensing Performances

**Figure 1:**
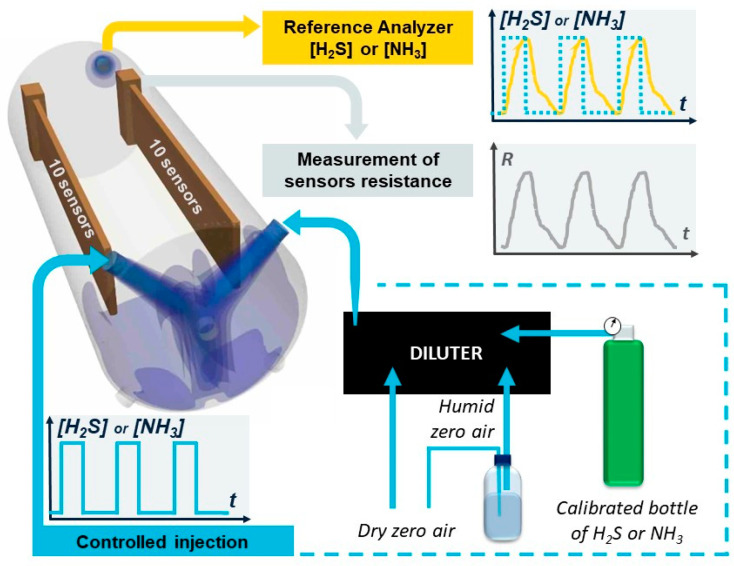
Experimental set-up to characterize the evolution of the resistance of 20 sensors in a controlled environment. The exposure chamber was maintained at a constant temperature (20–22 °C).

The gas detection performances of twenty sensors were simultaneously characterized in a controlled environment, using the set-up described in Figure 1. The sensors were connected to an electronic card and enclosed in a Teflon exposure chamber (0.5 L) maintained at room temperature (20–22 °C). During the entire experiment, the flow velocity (5 L/min), the H_2_S concentration and the humidity were controlled by the diluter Omicron technology OMI-SR042A-A, containing two mass flow controllers to dilute the calibrated gas bottles (flow ranges 100 sccm and 1000 sccm) and a third one dedicated to humidity regulation (flow range 20,000 sccm). The concentration of H_2_S inside the chamber and the variation of resistance of each sensor were measured in real time by an H_2_S Analyzer-Model T101 from TELEDYNE Envicontrol T series and the computer digital multimeter Keithley Agilent 34970A, respectively.

During the calibration of the sensors, each injection of mixture of humidity and H_2_S lasted 20 min. Desorption under humid filtered air lasted 60 min. The H_2_S concentration varied from 10 ppb to 1 ppm. The temperature and relative humidity, which corresponded to the amount of water in the air compared to the maximum amount that could possibly be held at a given temperature, were constant during the exposition/desorption cycles. The temperature was stabilized at 20–22 °C and the relative humidity was equal to 50% RH for the entire measurement, apart from the analysis of the effect of the humidity on the sensors.

The relative response (*R_r_*) given as a percentage is calculated according to Equation (1):

(1) $$R_{r}=\frac{R_{H2S}-R_{air}}{R_{air}}\cdot 100$$

with *R_H2S_* and *R_air_* the resistance of the sensor toward H_2_S and air, respectively [15]. Moreover, the limit of detection is calculated under 9 cycles of H_2_S injection at 167 ± 9 ppb by applying the following formula (Equation (2)):

(2) $$LOD=\frac{3\times \sigma \times \left[H_{2}S\right]}{\langle R_{min}\rangle }$$

with [*H_2_S*] the concentration of the gas measured by the analyzer, <*R_min_*> and *σ* the mean and the standard deviation of the relative response of the sensor for 9 cycles, respectively [26].

Next, the effect of humidity on the resistance of the sensors was evaluated by injecting air at different humidity levels for 2 h from 10 to 80% RH. According to the temperature and relative humidity sensor, Sensirion SHT25, stored in the exposure chamber, the temperature was maintained between 20 and 21 °C while the humidity varied (Figure S2 in the Supplementary Information). The resistance calculated corresponded to the mean of the resistance during the last hour of the cycle. Moreover, the sensors’ calibration toward H_2_S was performed under a constant humidity equal to 20%RH in order to characterize the impact of humidity on the response of the sensor.

Finally, the response to ammonia was studied by using the alternate injection of ammonia for 20 min at 574 ± 5 ppb and air for 60 min. The NH_3_ analyzer by laser spectroscopy LGR followed the concentration of ammonia in the chamber during the experiment.

## 3. Results and Discussion

### 3.1. Material Characterization

**Figure 2:**
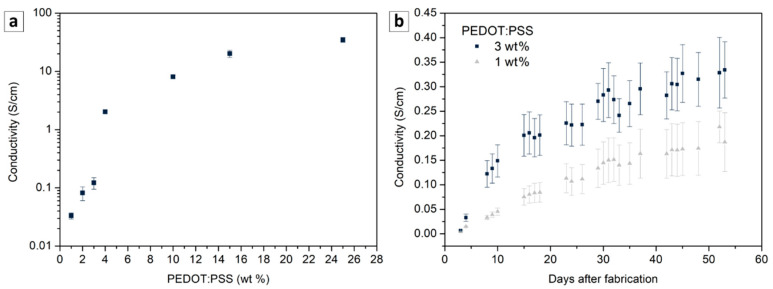
Conductivity of PAni-SnCl_2_-PEDOT:PSS composites. Evolution of the conductivity (measured one week after fabrication) as a function of PEDOT:PSS content (**a**) and as a function of time for two PEDOT:PSS content (**b**).

Known to be highly conductive [20], PEDOT:PSS was added to PAni-SnCl_2_ in order to facilitate the charge transport and to adapt the initial conductivity of the sensor to the requirements of the ohmmeter, working in the range of tens to 10^5^ ohms. More than a simple conductive additive, PEDOT:PSS can also increase the conductivity of PAni composites by protonating PAni thanks to the presence of protons in PSS (SO_3_^−^H^+^ groups) [27]. Indeed, the PEDOT:PSS used here featured a conductivity of 835 ± 86 S/cm, while the PAni-SnCl_2_ demonstrated a conductivity lower than 10^−5^ S/cm. As shown in Figure 2a, less than 4 wt% of PEDOT:PSS were enough to obtain conductivity fitting with the range of the measurement set-up. Only the blends with 1, 2 and 3 wt% of PEDOT:PSS were considered for the following characterizations given the fact that sensor made with more than 3 wt% showed a low gas detection performance (with a response several tens times lower).

**Figure 3:**
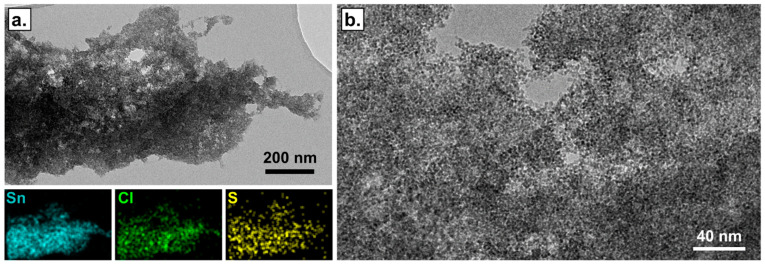
TEM images of PAni-SnCl_2_ composite containing 3 wt% of PEDOT:PSS observed at different scales before any contact with hydrogen sulphide (**a**) and (**b**). The elementary chemical mapping from TEM image (**a**) show a good dispersion of tin (Sn, blue) and chlorine (Cl, green) atoms and of sulphur (S, yellow) atoms carried only by PEDOT and PSS molecules.

As shown in the SEM images (Supplementary Information, Figure S3), the concentration of PEDOT:PSS in the range 1 to 3 wt% did not drastically influence the morphology of the film. All the samples presented a globular morphology with the presence of some microscopic pores (diameter about 1 µm). Next, TEM was employed to investigate more precisely the nanostructure of the composite (Figure 3). As shown on the elementary chemical mapping from the TEM image (Figure 3a), the materials showed a high level of uniformity at nanoscale with a good dispersion of SnCl_2_ in the polymers. In addition, the micrographs from different zones did not differ from each other. This result was confirmed by the nanoanalysis of different zones, which showed the same results. The representative EDS Spectra of the PAni-SnCl_2_-PEDOT:PSS 3 wt% is available in the Supplementary Information (Figure S4).

Subsequently, the stability of the blends was studied by measuring the conductivity of the film during 172 days at 21 °C under ambient conditions (with the relative humidity kept in the range 30 to 50%). While the pure PEDOT:PSS conductivity was almost unchanged during 6 months (i.e., loss of 1%), the blends showed a low and continuous increase in conductivity during the same period. For example, the conductivity of the PAni-SnCl_2_ blends with 3 wt% of PEDOT:PSS presented a high increase during the first week from (7 ± 1)·10^−3^ to (1.2 ± 0.3)·10^−1^ S/cm, followed by a continuous increase reaching (5.5 ± 0.2)·10^−1^ S/cm after 5 months (Figure 2b). This behavior seems to have resulted from the doping of the PAni over the time, mainly due to the presence of the SnCl_2_ and, to a lesser extent, to the presence of excessive PSS ions. Indeed, both species are known to act as doping agents [23,24,28] and the conductivity measured on the PAni-SnCl_2_ and PAni-PEDOT:PSS (3 wt%) increased by110% and 5%, respectively, after 28 days.

**Figure 4:**
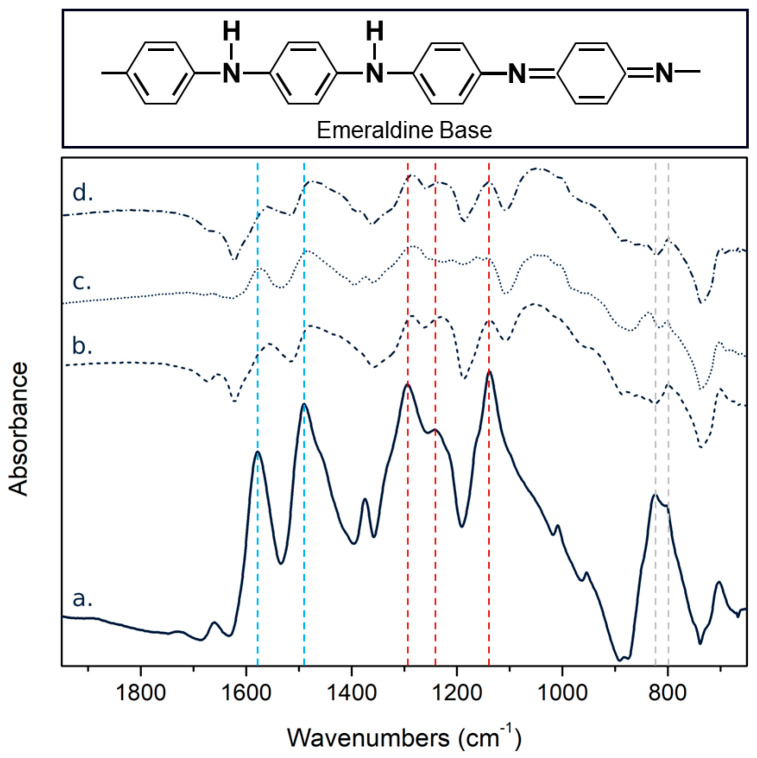
Chemical structure of the Pani’s emeraldine base and FTIR spectra of (**a**) PAni, (**b**) PAni-SnCl_2_, (**c**) PAni-PEDOT:PSS (3 wt%) and (**d**) PAni-SnCl_2_-PEDOT:PSS (3 wt%). The blue, red and grey lines correspond to the bands relative to the oxidation process, the protonation and the substitution of the aromatic rings, respectively.

FTIR spectroscopy was conducted on the PAni, PAni-SnCl_2_, PAni-PEDOT:PSS (3 wt%), and PAni-SnCl_2_-PEDOT:PSS (3 wt%) in order to confirm the interaction between the different components of the sensitive material. As shown in Figure 4a, the Pani spectra is representative of spectra from emeraldine from.. It presents two main absorption bands associated with the oxidation state of the polymer at 1578 and 1490 cm^−1^, three bands associated with its protonation at 1293, 1241 and 1138 cm^−1^ and one band, around 823 cm^−1^, relative to the substituent’s pattern on the benzenoid rings and corresponding to aromatic C-H out-of-plane deformation vibrations [29,30,31]. The bands at 1578 and 1490 were attributed to the stretching vibration of C=C in the quinoid (Q) and benzenoid (B) rings, respectively. The three bands associated with the protonation were attributed to the stretching mode of the secondary amine (C-N-C) [32] or imine (-N=) at 1293 cm^−1^ [33], to the stretching vibration in the polaron structure (C-N^+^ or C=N^+^) at 1241 cm^−1^ and to the stretching of the protonated imine at 1138 cm^−1^ [34].

Firstly, the oxidation degree of the PAni, which is linked to the relative amount of quinoid rings in the structure [35], was slightly increased by the addition of PEDOT:PSS and SnCl_2_. The ratio of the bands’ intensities, associated with the quinoid and benzenoid rings (I_Q_/I_B_), increased from 0.87 to 0.95 in presence of these two components (Table 1).

Secondly, the main absorption bands (except 1138 cm^−1^) suffered a red shift in the presence of PEDOT:PSS and, more specifically, in the presence of SnCl_2_. This phenomenon, which is often recognized as a sign of the protonation process [36,37,38], confirmed that both of the additives and most of the metal salt contributed to the doping of the PAni. The ability of SnCl_2_ to dope PAni is also supported by the increase of the relative intensity of the peak attributed to the polaron structure compared to the peak associated with the amine or imine groups (I_N_^+^/I_N_). Indeed, this ratio increased from 0.90 to 1.0 with the addition of SnCl_2_ (Table 1). 

Finally, the distribution of the bands between 1000 and 700 cm^−1^ changed as a function of the material composition, indicating the influence of the additives on the structure of the benzene ring. As observed on PAni doped with iron and copper ions [39], the peak at 800 cm^−1^ became stronger than the one at 823 cm^−1^ in the presence of SnCl_2_. Thus, the FTIR spectra confirmed the doping action of SnCl_2_ and the strong interactions between the components of the hybrid material.

### 3.2. Detetion Performance under 50% of Humidity

#### 3.2.1. Calibration

**Figure 5:**
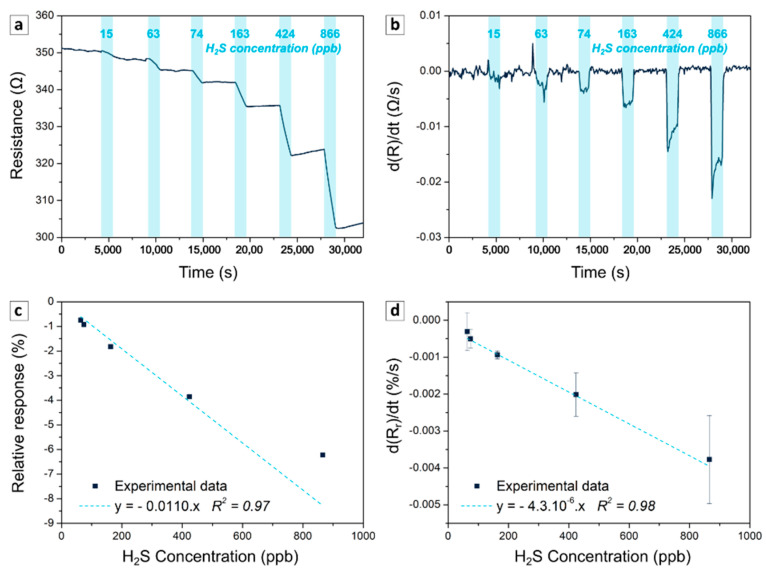
Response to hydrogen sulphide of sensor containing 3 wt% of PEDOT:PSS. Evolution of the resistance (**a**) and its derivative (**b**) as a function of time during cycles with increasing concentrations of hydrogen sulphide, from 15 to 866 ppb. Corresponding calibration curve expressed as the variation of the relative response (**c**) and the mean of its derivative (**d**) as a function of the concentration of hydrogen sulphide.

Figure 5a presents the typical response of the PAni-SnCl_2_-PEDOT:PSS blend to H_2_S, i.e., the decrease in resistance of the material with the injection of H_2_S. Indeed, H_2_S is known to react with metal chloride (MCl_2_) to form a metal sulphide (MS) and releases strong acid (HCl), which dopes the PAni and decreases the resistance of the polymer [16], as illustrated in the following equation (Equation (3)):

H_2_S + MCl_2_ → MS + 2HCl (3)

However, the reversibility was limited during the 60 min desorption. The metal chloride hybrids always demonstrated this typical behavior given the fact that the reaction with the gas led to the formation of a stable and conductive metal sulphide [17]. Moreover, even at a low concentration (63 ppb), the response of the sensor (around −0.5 to −0.8% depending on the concentration of PEDOT:PSS) was still appreciable, while only a few chemiresistive H_2_S sensors based on conductive polymers have shown responses below 100 ppb [40,41,42,43]. Moreover, the derivative of the signal showed an abrupt inflection at the injection of H_2_S gas and the return to zero at the end of the exposure, confirming the fast response of the sensors. The response time calculated here as the time needed for the derivative to reach 90% of its value was lower than 100 s in the range of 50–1000 ppb Figure 5b), which is close to the shortest response time of metal chloride hybrids [15]. Thus, PAni-SnCl_2_-PEDOT:PSS is highly sensitive and quick to respond to H_2_S present at the sub-ppm level.

Next, two methods were used to calibrate the sensor. Firstly, the calibration was performed by calculating the minimum of the relative response for each cycle. As shown in Figure 5c, the calibration curves presented a good linearity in the range 50 to 600 ppb. For the blends with 1, 2 and 3 wt% of PEDOT:PSS, the R-squares were 0.94 ± 0.04, 0.98 ± 0.01 and 0.97 ± 0.01, respectively and the mean sensitivity (slope of the curve) was about −11 ± 3, −11 ± 5 and −9 ± 3%/ppm, respectively. 

Secondly, to facilitate the use of the sensor in real applications, the calibration was performed by calculating the mean of the derivative of the relative response (d(R_r_)/dt expressed in %/s) at each cycle. Indeed, in real-time measurement, it was easier to calculate the derivative than to find the local minimum of the signal. As shown in Figure 5d, the calibration curves based on the derivative also demonstrated a good linearity in the range of 50 to 600 ppb, with an R-square of 0.84 ± 0.05, 0.94 ± 0.02 and 0.95 ± 0.03 for the samples with 1, 2 and 3 wt% of PEDOT:PSS, respectively. Each value was derived from the mean and the standard deviation calculated on at least three samples.

#### 3.2.2. Repeatability, Reproducibility, Stability

Beyond the detection performance of the sensors, their repeatability, reproducibility and stability are crucial.

**Figure 6:**
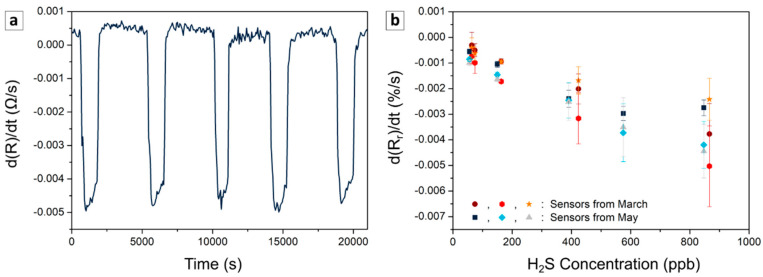
Repeatability and reproducibility of the sensors: derivative of the resistance as a function of time for five consecutive cycles of injection at 167 ± 9 ppb of H_2_S (**a**) and calibration curves based on the derivative of the relative response of two series of three different sensors (**b**). Each form represents different sensors produced in two batches (one in March and one in May). All the sensors contained 3 wt% of PEDOT:PSS and were studied one month after their fabrication.

The repeatability was evaluated through nine consecutive injections of 167 ± 9 ppb H_2_S. As shown in Figure 6a, the sensors demonstrated a good repeatability, with the limit of detection decreasing in line with the increase of PEDOT:PSS content. The materials with 1, 2 and 3 wt% of PEDOT:PSS presented a limit of detection around 82, 31 and 28 ppb, respectively. In the following section, the results focus on the blend PAni-SnCl_2_ containing 3 wt% of PEDOT:PSS, given its lower limit of detection.

Figure 6b presents the calibration curves of the sensors from a solution of 3 wt% of PEDOT:PSS prepared one month apart (three sensors per batch). Based on the calibration from the derivative, the sensitivity of the different sensors was in the range of [−6.5;−2.6]·10^−3^%/(s·ppm). These results confirm that the devices based on PAni-SnCl_2_-PEDOT:PSS presented reproducible detection behavior independently of the batch of production.

**Figure 7:**
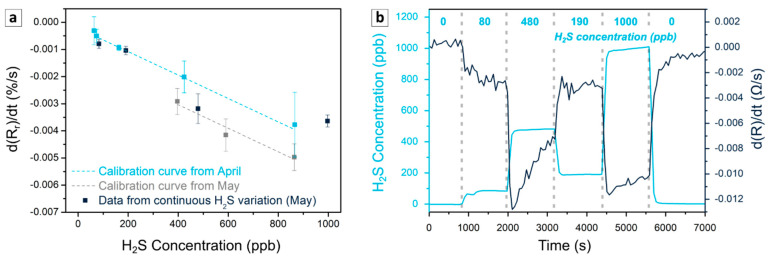
Signal variation of PAni-SnCl_2_ composites containing 3 wt% of PEDOT:PSS during the continuous variation of H_2_S concentration. (**a**) Comparison of the relative derivative from the continuous variation of H_2_S concentration with the calibration previously established. (**b**) Variation of the concentration given by analyzer and the derivative of the signal from the sensors as a function of time.

Next, the stability of the sensor was studied over one month by calibrating the sensors one and two months after their fabrication (Figure 7a). Even though the relative derivative signal presented a light shift for lower values over the time, the sensor sensitivity remained almost unchanged (about 0.6% changes).

#### 3.2.3. Use Case: Detection under Continuous Variation of H_2_S Concentration

To confirm the potential of the sensors for use in real applications, the sensors were studied through the random variation of the H_2_S concentration without desorption steps, in fresh air. As seen in Figure 7b, the derivative of the signal followed the variation of the H_2_S shown by the analyzer connected to the chamber. Next, the mean of the derivative was calculated for each step and was superposed into calibration curves. According to Figure 7a, the response of the sensors during the continuous variation of H_2_S fitted well with the calibration curves. This experiment at room temperature and atmosphere confirmed that the sensors can be used to quantify H_2_S concentration during continuous variation, after a suitable calibration based on the analysis of the derivative of the signal built from alternate cycles of H_2_S and air.

### 3.3. Impact of Interfering Species

#### 3.3.1. Humidity

**Figure 8:**
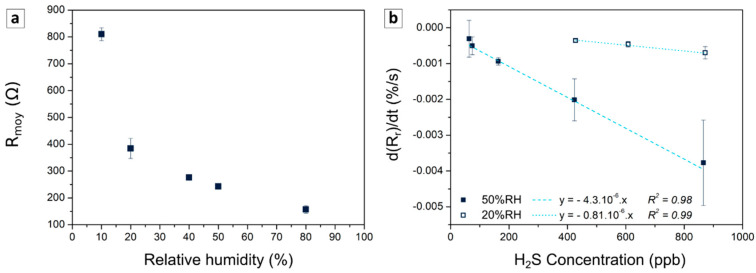
Impact of the humidity on the resistance of a sensor based on composite with 3 wt% of PEDOT:PSS under zero air (**a**). Impact of the humidity on the calibration curve expressed as the variation of the derivative as a function of the concentration of hydrogen sulphide at 50% and 20% of relative humidity (**b**).

H_2_S sensors can be used in environments with varying humidity. First, the effect of this parameter on the resistance of the material containing 3 wt% PEDOT:PSS was studied in air. As is often observed in PAni blends [44], the resistance tends to decrease along with an increase of relative humidity (Figure 8a). Indeed, water molecules adsorbed on the polymer release protons, which enhance the charge transport by ionic conduction and/or by increasing the doping level of the polymer [45,46]. The humidity leads to higher resistance changes than H_2_S at sub-ppm level and makes quantification difficult if it is not stable. Given the high resistance variation at low RH, the effect of humidity on the response of the sensor was studied by calibrating the sensor at 50 and 20% RH. The decrease of the humidity led to a drastic decrease in the sensitivity, which was divided by a factor higher than 5 (Figure 8b). Pani-based sensors are known to present better sensing properties at higher humidity due to their enhanced charge transfer in the presence of water [43,47]. However, further investigations are needed to understand the role of humidity in the sensing performance of those sensors more clearly. Thus, humidity control or compensation are needed to apply these materials to real applications, given its high impact on the resistance and the sensitivity of the sensors.

#### 3.3.2. Oxidative Gas

**Figure 9:**
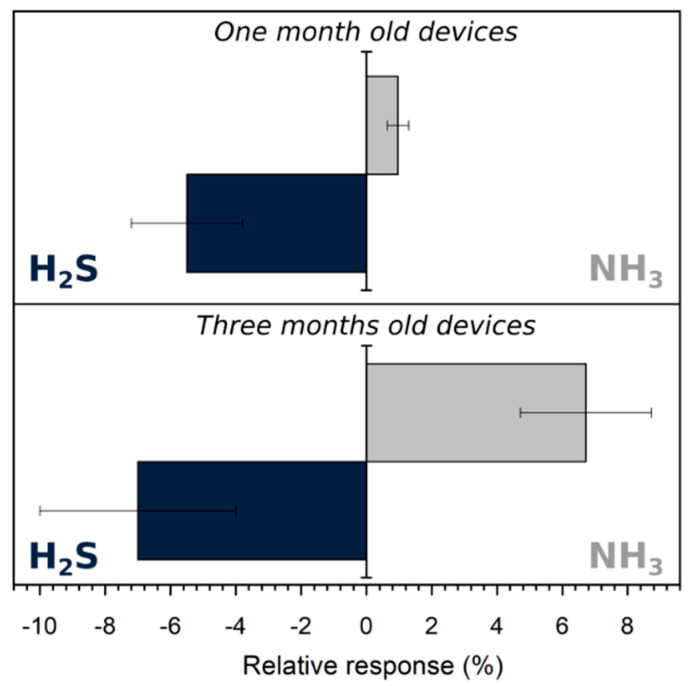
Relative response of devices one (**up**) and three (**down**) months old to 574 ± 5 ppb of H_2_S (left and blue) and NH_3_ (right and grey).

Ammonia can be a crucial interfering specie given the fact that PAni blends are known to be highly efficient ammonia gas sensors [48] and that this gas can be present concurrently with hydrogen sulphide in the field (e.g., during agricultural spreading, the decomposition of Sargassum seaweed, etc.). Therefore, three sensors based on 3 wt% PEDOT:PSS blends were exposed to 574 ± 5 ppb of H_2_S and NH_3_, one and three months after their fabrication (Figure 9). Unlike H_2_S, NH_3_ induced an increase of t resistance given its reducing nature. Indeed, in the presence of NH_3_, the amount and the mobility of the charge carrier reduced due to the removal of a proton from PAni to form ammonium cation (NH_4_^+^) [48], as illustrated in the Equation (4):

PAniH^+^ + NH_3_ = PAni + NH_4_^+^ (4)

Moreover, the one-month-old samples presented an absolute relative response to H_2_S that was six times higher than it was to NH_3_, while the relative responses were −6 ± 2% and 1.0 ± 0.3%, respectively. Thus, H_2_S sensing was dominant on the fresh device. However, two months later, when the response to H_2_S (−7 ± 3%) was almost unchanged, the response toNH_3_ increased drastically from 1.0 ± 0.3 to 7 ± 2%. As seen previously (Figure 2b), the polymer tended to be more conductive with time when it was stored in room atmosphere. As discussed previously, the increase of conductivity can be linked to an increase of the doping ratio of PAni, which leads to an expansion of the numbers of active sites able to react with NH_3_. In this way, the device became more sensitive to ammonia by ageing. Time did not have the same effect on H_2_S detection given the fact that for this gas, the detection performances of the composite should mainly depend on the ability of SnCl_2_ to release HCl in the presence of H_2_S but not on the doping state of PAni, which was not a limiting factor.

## 4. Conclusions

For the first time, H_2_S sensors based on metal chloride hybrids were studied at sub-ppm level. Beyond the sensing performance of the devices, the morphology and chemical structure of the PAni-SnCl_2_-PEDOT:PSS blends used as sensitive surfaces were confirmed by SEM, TEM, EDS and FTIR analysis. Showing a low detection limit, rapidity, repeatability, reproducibility and stability over one month, sensors based on this material demonstrated high potential for H_2_S detection despite the irreversibility of their response. Indeed, this study has shown how calibration based on the derivative of the signal can overcome the irreversible response of the sensors and make them able to quantify the concentration of the target gas, even during continuous variation of the analyte concentration. Experiments with ammonia and different levels of relative humidity highlighted the limitations of those devices given the growing impact of the ammonia with ageing and the continuous impact of the humidity on the response of the sensors. Finally, more investigations are needed to understand the effects of humidity and time in order to optimize those materials and make them operational for long-term applications in the field.

## Figures and Tables

**Table 1:** FTIR spectral data of the different composites based on PAni (cm^−1^). The blends with PEDOT:PSS contained 3 wt% of this polymer.

Samples	PAni	PAni-SnCl_2_	PAni-PEDOT:PSS	PAni-SnCl_2_-PEDOT:PSS
C=C (Quinioid, Q)	1578	1556	1575	1559
C=C (Benzenoid, B)	1490	1478	1484	1476
C-N or –N=	1293	1284	1284	1287
C-N^+^ or C=N^+^	1241	1230	-	1236
-NH^+^=	1138	1138	1141	1141
N=Q=N, SO_3_^−^	-	1055	1052	1053
C-H out-of-plane for different mode of aromatic rings	823	800	835/801	800
Ratio I_Q_/I_B_	0.87	0.92	0.93	0.95
Ratio I_N_^+^/I_N_	0.90	1.0	-	0.97

I_Q_, I_B_, I_N_^+^, I_N_: intensity of the quinoid band, the benzenoid band, the band relative to the polaron (~1240 cm^−1^) and the band relative to the secondary amine or imine (~1290 cm^−1^).

## Supplementary Materials

The following are available online at https://www.mdpi.com/article/10.3390/s21196529/s1. Table S1: Composition of the solution of polymer composites. Figure S1: Preparation of the sensors. Figure S2: Example of experiment under changing humidity and stable temperature. Humidity and temperature were directly measured in the chamber, where the sensors were characterized under different controlled environments. Figure S3. SEM images of PAni-SnCl2 composites containing 1 wt% (a), (b) and 3 wt% (c), (d) of PEDOT:PSS. Figure S4. EDS nanoanalyses characteristic of PAni-SnCl2-PEDOT:PSS (3 wt%) samples.
