# Supplementary material for: Conductive Polymer Composites for Hydrogen Sulphide Sensors Working at Sub-PPM Level and Room Temperature
Source: Sensors (Basel). 2021 Sep 30;21(19):6529. doi: 10.3390/s21196529 (PMC8512851; doi:10.3390/s21196529)
Supplement: Supplementary file 1 [file sensors-21-06529-s001.zip › sensors-1401075-supplementary.pdf]

Supplementary information

PEDOT :PSS content	PAni	$SnCl_2$	$(PEDOT:PSS/EG)_{aq}$
wt%		mg	
0	40.3	62.4	**0**
1	40.0	62.5	102.8
2	40.0	62	208.7
3	40.0	62.3	316.8
4	40.0	62.0	435.4
10	39.8	61.9	1133
15	39.7	61.5	1800
25	39.7	62.7	3400

Table S1. Composition of the solution of polymer composites.

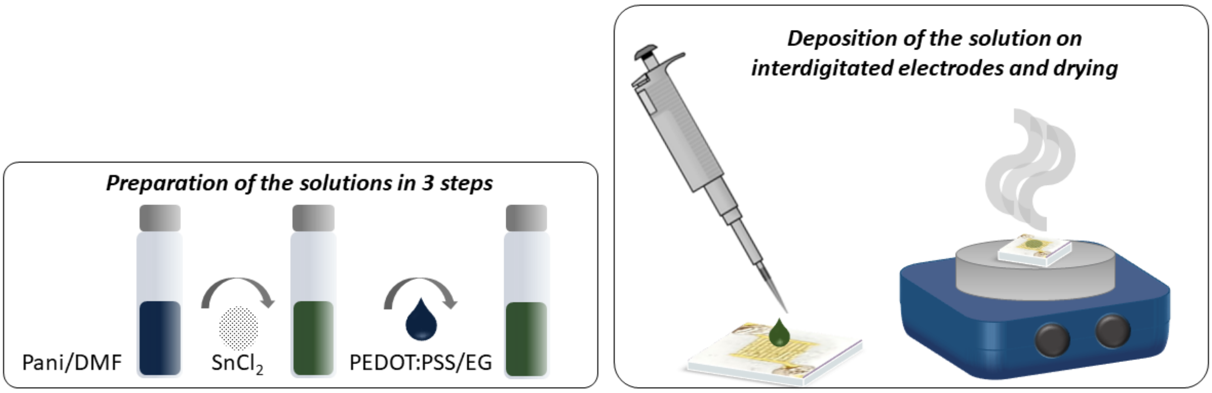

Figure S1. Preparation of the sensors

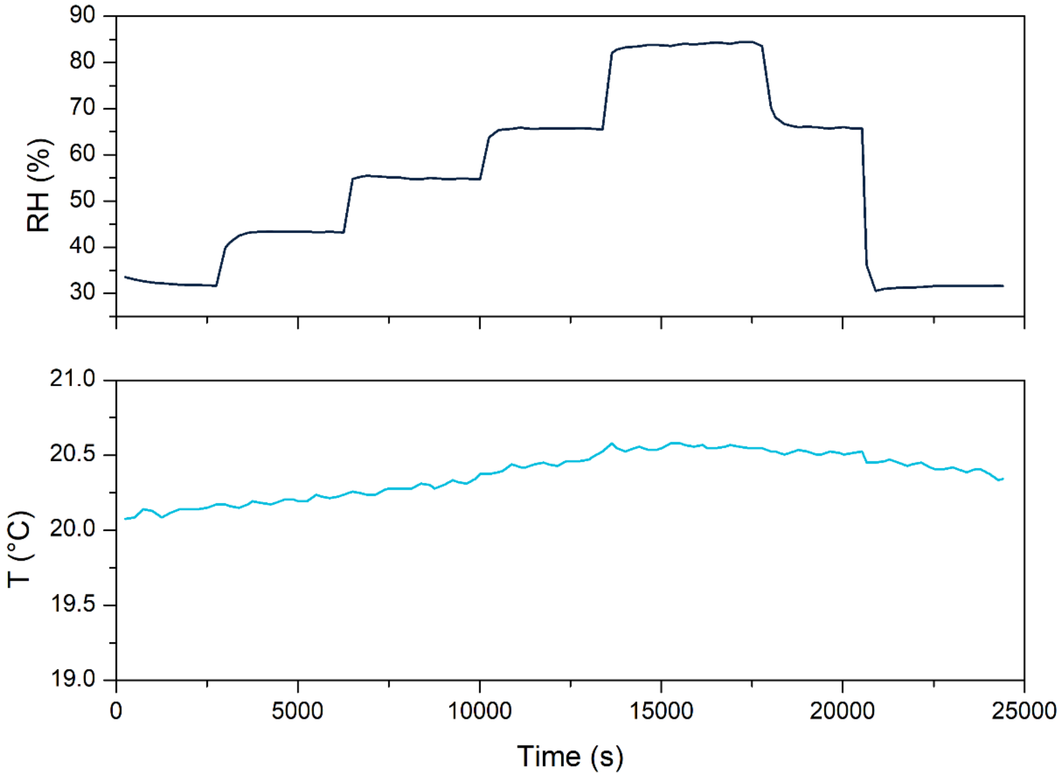

Figure S2. Example of experiment under changing humidity and stable temperature. Humidity and temperature are directly measured in the chamber where the sensors are characterized under different controlled environments.

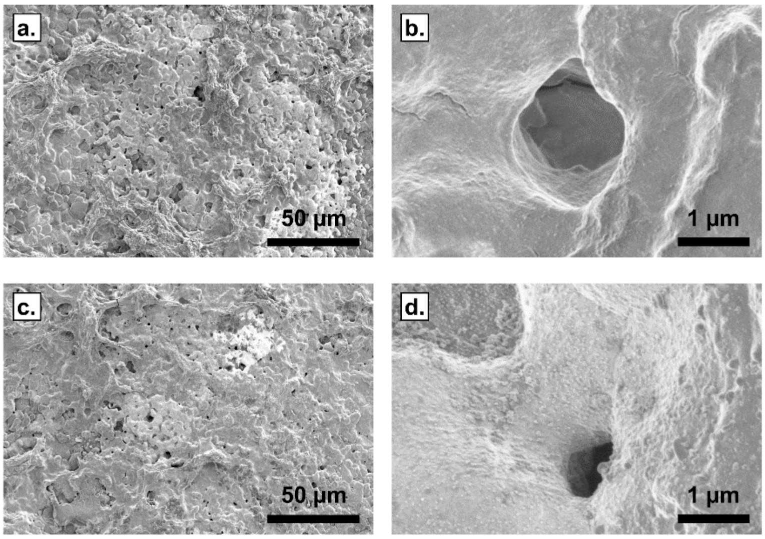

Figure S3. SEM images of PAni-$SnCl_2$ composites containing 1 wt% (a), (b) and 3 wt% (c), (d) of PEDOT:PSS.

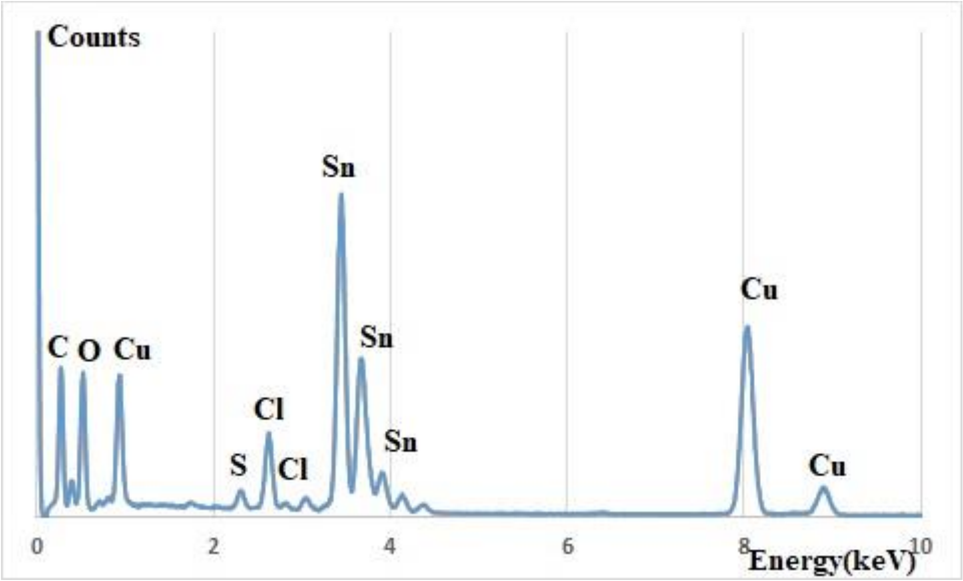

Figure S4. EDS nanoanalyses characteristic of PAni-$SnCl_2$-PEDOT:PSS (3 wt%) samples. The copper line is characteristic of the sample holder grid.
